# Clinical practice variability among paediatric interventional cardiologists assessing pulmonary arteriovenous malformations

**DOI:** 10.1017/S1047951125110019

**Published:** 2025-10-10

**Authors:** Joshua Fields, Jared Boon, Osama Aldoss, Susan R. Foerster, Todd M. Gudausky, Stephen B. Spurgin, Andrew D. Spearman

**Affiliations:** 1 Department of Pediatrics, Division of Cardiology, Medical College of Wisconsin, Children’s Wisconsin Herma Heart Institute, Milwaukee, WI, USA; 2 Department of Pediatrics, Division of Quantitative Health Sciences, Medical College of Wisconsin, Milwaukee, WI, USA; 3 Cardiovascular Research Center, Medical College of Wisconsin, Milwaukee, WI, USA; 4 Department of Pediatrics, Division of Cardiology, University of Texas Southwestern Medical Center, Dallas, TX, USA

**Keywords:** pulmonary arteriovenous malformation, single ventricle, Glenn, Fontan, hepatic factor, CHD, echocardiography

## Abstract

**Background:**

Single ventricle pulmonary arteriovenous malformations are poorly understood and variably assessed in published literature. To improve our understanding of single ventricle pulmonary arteriovenous malformations and facilitate multi-centre studies, it will be necessary to have uniform clinical practice patterns among paediatric heart institutions.

**Objectives:**

The aim of this study was to assess paediatric interventional cardiologists’ clinical perspectives and practice patterns for diagnosing single ventricle pulmonary arteriovenous malformations.

**Methods:**

We surveyed paediatric interventional cardiologists using the Congenital Cardiovascular Interventional Consortium listserv. A single survey was distributed electronically with two subsequent reminder emails. Voluntary participants completed the anonymous survey electronically via RedCap.

**Results:**

Among 253 Congenital Cardiovascular Interventional Consortium members, a total of 55 (21.7%) paediatric cardiology interventional attending physicians completed the survey. There was near unanimity (98%) that pulmonary arteriovenous malformations develop due to lack of hepatic vein blood flow to the lungs; however, there was wide variation among practice patterns. A minority (20%) of respondents perform bubble contrast echocardiograms (bubble studies) more than half the time pre-Fontan, whereas many (31%) almost never (< 5% of cases) perform bubble studies pre-Fontan. Most respondents reported that they did not perform bubble studies because results do not impact clinical decision making pre-Fontan (56%) or post-Fontan (60%). Many respondents (49%) do not have a typical volume of agitated saline that they inject for bubble studies.

**Conclusions:**

Clinical practice patterns vary widely among paediatric cardiology interventionalists. A standardised clinical approach, new diagnostic tools, or both are needed to standardise our field’s approach to diagnosing, studying, and treating single ventricle pulmonary arteriovenous malformations.

## Introduction

Pulmonary arteriovenous malformations are vascular malformations that universally develop in patients with palliated single ventricle CHD.^1-5^ Single ventricle pulmonary arteriovenous malformations were first reported more than 50 years ago when they were observed to develop after Glenn palliation.^6,7^ Despite the long-standing clinical recognition, single ventricle pulmonary arteriovenous malformations remain poorly understood with no known medical treatments.

Contrast echocardiograms with agitated saline (bubble echos or bubble studies) are a commonly used clinical tool to assess for pulmonary arteriovenous malformations in single ventricle circulation and in hereditary forms of AVMs, such as hereditary haemorrhagic telangiectasia.^8-13^ Clinical practice guidelines for hereditary haemorrhagic telangiectasia were updated in 2020 and recommended screening for pulmonary arteriovenous malformations with bubble echocardiograms, but no such guidelines currently exist for single ventricle pulmonary arteriovenous malformations.^14,15^ In fact, existing guidelines for hereditary haemorrhagic telangiectasia cannot be directly adapted for use in patients with single ventricle circulation due to variable and complex single ventricle circulation. For example, peripheral vein injections used in hereditary haemorrhagic telangiectasia can lead to false positive or false negative findings in single ventricle circulation due to veno-venous collaterals or streaming/unequal pulmonary blood flow, respectively. Ultimately, uniform clinical practice patterns in single ventricle CHD are necessary to facilitate multi-centre studies of this relatively small and heterogenous patient population. Thus, the objective of this study was to determine the clinical perspectives and practice patterns among paediatric interventional cardiologists who directly diagnose single ventricle pulmonary arteriovenous malformations.

## Methods

### Survey

We electronically surveyed paediatric interventional cardiologists using the Congenital Cardiovascular Interventional Consortium listserv. The Congenital Cardiovascular Interventional Consortium listserv is an email communication for Congenital Cardiovascular Interventional Consortium members to collaborate on clinical and research topics related to CHD. Congenital Cardiovascular Interventional Consortium members include paediatric interventional cardiologists predominantly from North America but also from South America and Europe. The Congenital Cardiovascular Interventional Consortium database is currently housed at Joe DiMaggio Children’s Hospital in Hollywood, Florida, USA. A single survey was distributed electronically with two subsequent reminder emails. Voluntary participants completed the anonymous survey electronically via RedCap. This survey study was reviewed and approved by the Institutional Review Board at the Medical College of Wisconsin. An Institutional Review Board-approved informational letter was included in the listserv emails and approved by the Institutional Review Board in place of a documented informed consent.

To identify the experience level of survey respondents, we collected demographic data on clinical experience and clinical practice location. To identify perspectives about the aetiology of single ventricle pulmonary arteriovenous malformations, we collected data on specific variables involved in single ventricle pulmonary arteriovenous malformation pathogenesis. To identify overall conceptual approaches to single ventricle pulmonary arteriovenous malformations (i.e., whom and when to test), we collected data on the frequency of bubble study testing pre- and post-Fontan, as well as the rationale for performing or not performing bubble studies. Lastly, to identify potential variability in the technical aspects of assessing single ventricle pulmonary arteriovenous malformations, we collected data on technical aspects of bubble study testing and pulmonary vein oximetry testing.

### Statistical analysis

Data are expressed as median and interquartile range for continuous data and *n* (%) for categorical data unless otherwise stated. Analyses were performed using GraphPad Prism 10 (GraphPad Software, San Diego, CA).

## Results

### Respondent demographics

A total of 55 respondents completed the voluntary survey, which was sent to a list of 253 registered listserv members for the Congenital Cardiovascular Interventional Consortium, yielding a 21.7% response rate. Demographics, including years of experience as an attending paediatric interventional cardiologist, number of Glenn or Fontan catheterisations performed annually, and current practice location, are summarised in Table 1.

### Aetiology of single ventricle pulmonary arteriovenous malformations

Survey respondents overwhelmingly (54/55, 98.2%) believe that single ventricle pulmonary arteriovenous malformations develop due to a lack of hepatic vein blood flow to the lungs (i.e., lack of hepatic factor) (Table 2). The single respondent who did not select the hepatic factor response selected non-pulsatile flow as the causative factor in single ventricle pulmonary arteriovenous malformations. Multiple answers were allowed with this question, yet only a minority (8/55, 14.5%) selected more than one causative factor. Of the potential additional factors, non-pulsatile flow was the most common answer (7/55, 12.7%). A small minority (6/55, 10.9%) selected both lack of hepatic factor and non-pulsatile flow. The single respondent who selected “other” identified lack of hepatic venous blood flow and self-reported that pulmonary arteriovenous malformations form due to “other unknown factors.”

### Conceptual considerations for assessing single ventricle pulmonary arteriovenous malformations

There was significant variability among respondents in various aspects of their conceptual approaches for assessing single ventricle pulmonary arteriovenous malformations (Table 3). In pre-Fontan catheterisations, a minority of respondents routinely perform bubble studies in over 50% of cases (11/55, 20.0%). In contrast, a larger proportion (17/55, 30.9%) perform pre-Fontan bubble studies in < 5% of cases. Among those who perform studies in < 5% of cases pre-Fontan, most responded (9/16, 56.3%) that bubble studies pre-Fontan do not impact clinical making. An additional open response commented that “AVMs large enough to be clinically significant are generally evident by angio. If small enough to need a bubble study, they don't impact decisions.” No respondents reported safety concerns about performing bubble echos.

Similar to pre-Fontan catheterisations, a minority of respondents routinely perform bubble studies in over 50% of cases post-Fontan (8/55, 14.6%). A similar proportion (10/55, 18.2%) perform post-Fontan bubble studies < 5% of cases. Surprisingly, though, a larger proportion of respondents (22/55, 40.0%) perform bubble studies in 5–24% of pre-Fontan cases. Among those who perform studies in < 5% of cases post-Fontan, most respondents (6/10, 60.0%) similarly responded that bubble studies post-Fontan do not impact clinical decision making. An open response commented that “sometimes [perform bubble studies] to assess if Fontan has helped AVMs regress, but for the most part rely on other data to determine pulmonary arteriovenous malformations.” Similar to pre-Fontan, no respondents reported safety concerns about performing bubble echos.

### Technical considerations for assessing single ventricle pulmonary arteriovenous malformations

**Figure 1.**
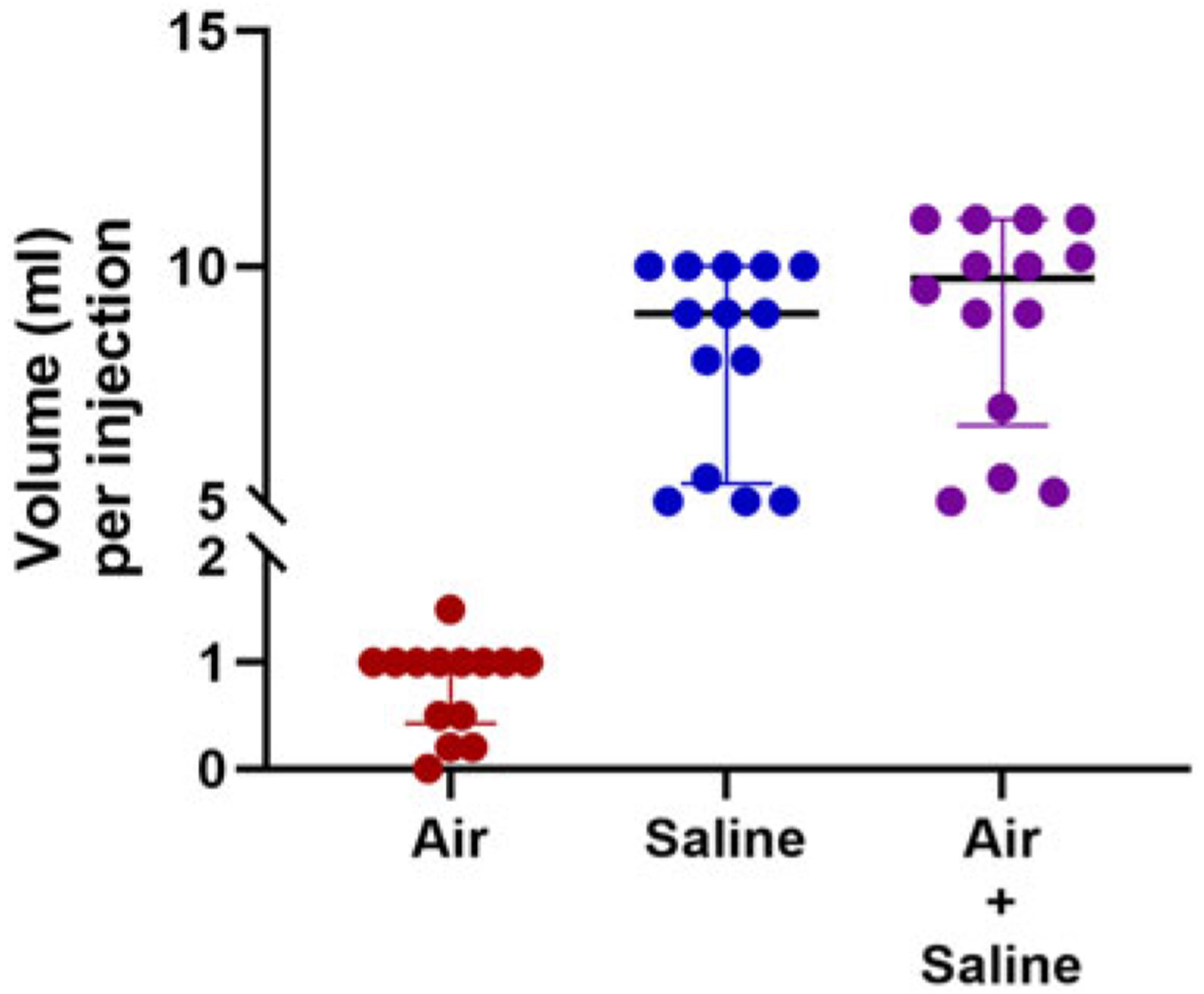
Volumes of air and saline used by respondents who self-reported using the same volume of air and saline for all bubble studies. Scatter plot showing each response with median and interquartile ranges.

When performing bubble studies in patients with single ventricle circulation, most, but not all, respondents (45/53, 84.9%) inject agitated saline separately into each branch of the pulmonary artery (Table 4). There was pronounced variation among respondents in the use of agitated saline injection for bubble studies (Table 4, Figure 1). A minority of respondents (15/53, 28.3%) reported using the same volume of air and saline for bubble study injections in all tests, but there was still variability among this group in the specific volumes used (Figure 1). Almost half of the respondents (26/43, 49.1%) reported that the volume of agitated saline they use for bubble studies varies from test to test. Factors influencing the variable amount of agitated saline included expected factors such as patient age and patient size; however, there were also unexpected factors influencing the volume of agitated saline, such as “available equipment” and “enough to make sure it’s not a false negative.”

Beyond technical considerations of bubble studies, we also assessed technical considerations for measuring pulmonary vein oxygenation (Table 4). Nearly all respondents measure pulmonary vein saturations pre-Fontan (50/55, 90.9%), but there is variability in where and how. More than half (28/50, 56.0%) report collecting upper and lower pulmonary vein samples pre-Fontan, whereas a large proportion (21/50, 42.0%) report collecting whichever vein is most easily accessible. Lastly, most respondents (30/50, 60.0%) only collect pulmonary vein samples under baseline FiO_2_ conditions.

## Discussion

In this survey of paediatric cardiology interventionalists, there is strong consensus about the aetiology of single ventricle pulmonary arteriovenous malformations, but there are pronounced differences in the conceptual and technical approaches for diagnosing single ventricle pulmonary arteriovenous malformations. These diagnostic differences highlight potential challenges in performing multi-institutional studies to improve our understanding of single ventricle pulmonary arteriovenous malformations or potential future clinical trials treating single ventricle pulmonary arteriovenous malformations.

Previous publications have hypothesised that single ventricle pulmonary arteriovenous malformations develop from lack of hepatic vein blood flow to the pulmonary vasculature (the so-called hepatic factor hypothesis), lack of pulsatile flow, or a combination of the two physiologic variables.^1,3,5,16-22^ While these variables have been given relatively equal weight in previously published literature, there was strong consensus among our respondents (98.2%) that single ventricle pulmonary arteriovenous malformations develop due to a lack of hepatic factor perfusion to the lungs, and only a minority (10.9%) selected both hepatic factor and non-pulsatile flow. Thus, the hepatic factor hypothesis is the current era consensus for single ventricle pulmonary arteriovenous malformation aetiology.

There are numerous guidelines in our field for follow-up and testing recommendations for CHD; however, our field does not yet have guidelines for when and how to assess single ventricle pulmonary arteriovenous malformations. In contrast, there are clear recommendations to screen and re-screen for pulmonary arteriovenous malformations with bubble echocardiography in patients with hereditary AVMs (i.e., hereditary haemorrhagic telangiectasia) and even detailed methodology for how to technically perform bubble echocardiograms in this patient population.^11,14,15,23^ Specifically, a review published in Journal of American Society of Echocardiography in 2015 recommended to perform bubble echos using 8 ml saline, 1 ml air,1 ml blood, and then subsequently inject 5 ml of this freshly agitated saline within 3 seconds in the antecubital vein.^11^ This approach differs slightly from the 2014 guidelines for cardiac sonographers published in the same journal with recommendations to use 8 ml saline agitated with 0.5 ml room air injected through a forearm or hand vein (no specification of blood, volume of injection, or rate of injection).^24^ Importantly, these recommendations differ from single ventricle pulmonary arteriovenous malformation assessment where most assessments are performed in the catheterisation lab with direct injection into branch pulmonary arteries. We propose, based on our survey results and previously published protocols, to perform single ventricle pulmonary arteriovenous malformation bubble echos in each lung by agitating 9 ml saline with 1 ml air and injecting 5–10 ml of agitated saline within 3 seconds with a catheter positioned in the proximal aspect of each branch pulmonary artery.

Many respondents in our survey indicated that their resistance to diagnostic testing was because bubble studies do not impact clinical decision making. In other words, it may be currently futile to diagnose single ventricle pulmonary arteriovenous malformations because we lack medical therapies for treating single ventricle pulmonary arteriovenous malformations. Despite this perspective, recent studies have identified potential molecular pathways that may be involved in single ventricle pulmonary arteriovenous malformation pathogenesis.^25,26^ Previous and current research groups have also developed animal models that effectively phenocopy single ventricle pulmonary arteriovenous malformations.^27-33^ Thus, research into single ventricle pulmonary arteriovenous malformations is progressing, identification of therapeutic targets in animal models is feasible, and clinical trials may realistically begin in the near future.

This cross-sectional survey has several limitations that are inherent with electronic survey studies. Foremost, we are limited by our small sample size with potential for selection bias. Our respondents are all paediatric cardiology interventional physicians, with most respondents having > 15 years of clinical experience as a cardiac interventional attending physician; however, responses may differ among paediatric cardiology sub-specialists who may refer patients for cardiac catheterisation. Additionally, despite providing opportunities for free-text responses, our survey is at risk for response bias.

In conclusion, our survey demonstrates that clinical practice patterns vary widely among paediatric interventional cardiologists. A standardised clinical approach, new diagnostic tools,^34^ or both are needed to advance our field’s approach to diagnosing, studying, and potentially treating single ventricle pulmonary arteriovenous malformations.

## Figures and Tables

**Table 1.** Respondent demographics

Years as a paediatric cardiac interventional attending	N (%)
< 5 years	6 (10.9)
5–15 years	16 (29.1)
> 15 years	33 (60.0)
Number of annual catheterisations performed on patients with Glenn or Fontan circulation	
< 10 cases	1 (1.8)
10–20 cases	18 (32.7)
> 20 cases	36 (65.5)
Location of current clinical practice	
North America	49 (89.1)
South America	2 (3.6)
Europe	4 (7.3)

N= number of respondents (percent).

**Table 2.** Survey responses—aetiology of single ventricle PAVMs

Question 1: I believe that PAVMs form due to … (select all that apply)	*N* = 55
Lack of hepatic vein blood flow to the lung (i.e., lack of hepatic factor)	54 (98.2)
Non-pulsatile pulmonary blood flow	7 (12.7)
Pathogenic factor(s) in SVC blood	2 (3.6)
Other	1 (1.8)

N= number of respondents (percent).

* 64 total responses from 55 survey respondents with 8/55 respondents (14.5%) checking more than one row (i.e., multifactorial aetiology).

**Table 3.** Survey responses—conceptual considerations for assessing single ventricle PAVMs

Question 1: Do you perform bubble studies pre-Fontan?	*N* = 55
Almost always (> 75%)	6 (10.9)
Frequently (50–75%)	5 (9.1)
Sometimes (25–49%)	13 (23.6)
Rarely (5–24%)	14 (25.5)
Almost never (< 5%)	17 (30.9)
Question 2: I perform bubble studies pre-Fontan for the following reasons: (select all that apply)	*N* = 37
Identify a potential cause of hypoxaemia	37 (100.0)
Identify a treatment target (ex: instruct surgeons for Fontan baffle placement)	7 (18.9)
Requested by the referring provider	4 (10.8)
Research interest	1 (2.7)
Other	3 (8.1)
Question 3: I almost never perform bubble studies pre-Fontan for the following reasons:	*N* = 16
Bubble studies pre-Fontan do not give accurate data	4 (25.0)
Bubble studies pre-Fontan do not impact clinical decision making	9 (56.3)
Not requested by referring provider	3 (18.8)
Other	4 (25.0)
Question 4: Do you perform bubble studies post-Fontan?	*N* = 55
Almost always (> 75%)	4 (7.3)
Frequently (50–75%)	4 (7.3)
Sometimes (25–49%)	15 (27.3)
Rarely (5–24%)	22 (40.0)
Almost never (< 5%)	10 (18.2)
Question 5: I perform bubble studies post-Fontan for the following reasons: (select all that apply)	*N* = 44
Identify a potential cause of hypoxaemia	43 (97.7)
Identify a treatment target (ex: creation of arteriovenous fistula)	12 (27.3)
Requested by the referring provider	4 (9.1)
Research interest	2 (4.5)
Other	0 (0.0)
Question 6: I almost never perform bubble studies post-Fontan for the following reasons:	*N* = 10
Bubble studies post-Fontan do not give accurate data	1 (10.0)
Bubble studies post-Fontan do not impact clinical decision making	6 (60.0)
Not requested by referring provider	2 (20.0)
Other	2 (20.0)

N= number of respondents (percent).

**Table 4.** Survey responses—echnical considerations for assessing single ventricle PAVMs

Bubble study techniques	
Question 1: When performing bubble studies, do you routinely inject agitated saline separately in each branch pulmonary artery?	*N* = 53
Yes	45 (84.9)
No	8 (15.1)
Question 2: When performing bubble studies, do you have a typical volume of agitated saline that you inject?	*N* = 53
Yes, always the same volumes of air and saline	15 (28.3)
Yes, always the same volume of saline but no specific volume of air	12 (22.6)
No, the volume varies	26 (49.1)
Oximetry techniques	
Question 3: Do you obtain pulmonary vein saturations pre-Fontan ?	*N* = 55
Yes	50 (90.9)
No	5 (9.1)
Question 4: When obtaining pulmonary vein saturations, where do you typically collect blood samples?	*N* = 50
Upper vein only	1 (2.0)
Lower vein only	0 (0.0)
Both upper and lower veins	28 (56.0)
Either upper or lower vein, whichever vein is most easily accessible	21 (42.0)
Question 5: When collecting pulmonary vein samples pre-Fontan , do you routinely collect blood under multiple conditions?	*N* = 50
No, only baseline FiO2	30 (60.0)
Yes, baseline + 100% O2	7 (14.0)
Yes (other)	13 (26.0)

N= number of respondents (percent).
